# LB1531A. Administration of abatacept, reduces mortality in hospitalized patients with COVID pneumonia. Results of a randomized controlled trial.

**DOI:** 10.1093/ofid/ofac492.1884

**Published:** 2022-12-15

**Authors:** Emily R Ko, Kevin J Anstrom, Anne Lachiewicz, Reynold Panettieri, Martin Maillo, Jane A O'Halloran, Cynthia Boucher, Michael A Maldonado, Soju Chang, Samuel Bozzette, William G Powderly

**Affiliations:** Duke University School of Medicine, Durham, Durham, NC; University of North Carolina School of Public Health, Chapel Hill, North Carolina; University of North Carolina, Chapel Hill, North Carolina; Rutgers University, New Brunswick, New Jersey; Sanatorio Diagnostico, Santa Fe, Santa Fe, Argentina; Washington University School of Medicine, St Louis, Missouri; National Center for Advancing Translational Sciences, Bethesda, Maryland; Bristol Myers Squibb, Princeton, New Jersey; National Center for Advancing Translational Sciences, Bethesda, Maryland; National Center for Advancing Translational Sciences, Bethesda, Maryland; Washington University School of Medicine, St Louis, Missouri

## Abstract

**Background:**

Severe COVID-19 infection is characterized by a dysregulated hyperinflammatory state that contributes to morbidity and mortality. Immunomodulatory therapy has been shown to improve outcomes. We investigated if abatacept, CTLA-4-Ig, a selective costimulation modulator, provides additional benefit when added to standard of care.

**Methods:**

We conducted a double-blind, randomized, placebo-controlled trial evaluating abatacept (given as a single infusion of 10mg/kg, to a maximum of 1000 mg) compared to standard of care (including remdesivir and dexamethasone) in patients hospitalized with COVID-19 pneumonia. The primary outcome was median time to recovery by day 29. Key secondary endpoints included 28-day mortality.

**Results:**

A total of 1019 patients received an infusion (509 assigned to abatacept and 510 to placebo), constituting the analyzed modified intention-to-treat cohort. The mean age 54.9 years (SD 14.65), 60.5% were male, 44.2% Hispanic or Latino and 13.7% black. Patients were evenly matched in terms of severity of illness, and comorbidities. Participants randomized to abatacept did not show a statistically significant difference in the primary endpoint with a recovery rate ratio of 1.135 (95% CI 0.996-1.294, p=0.057) compared to placebo. The median (IQR) time to recovery was 9 days (8, 10) for both groups. The 28-day mortality in the abatacept arm was 11.0% and in control arm 15.0% (OR 0.62 (95% CI 0.41, 0.94)), with a 37.8% lower odds of dying in patients receiving abatacept. The improvement in mortality was demonstrated for patients requiring low or high flow O2 at baseline but was not seen in patients who required mechanical ventilation or ECMO at time of randomization. Subgroup analysis identified the strongest effect in those with baseline CRP >75mg/L, age >65 and diabetics. Safety data demonstrated slightly lower risk of adverse events. Rates of secondary infections were similar (abatacept 16.1% and placebo 14.3%).

**Conclusion:**

Although single-dose IV abatacept did not demonstrate statistically significant improvement in time to recovery, it did show a substantial reduction in 28-day mortality compared to standard of care.

**Disclosures:**

**Reynold Panettieri, Jr., MD**, AstraZeneca: Advisor/Consultant|AstraZeneca: Grant/Research Support|AstraZeneca: Honoraria|Equillium: Grant/Research Support|Genentech: Advisor/Consultant|Genentech: Grant/Research Support|Janssen: Grant/Research Support|MedImmune: Grant/Research Support|Merck: Honoraria|Novartis: Grant/Research Support|Origo: Grant/Research Support|RIFM: Advisor/Consultant|RIFM: Grant/Research Support|Sanofi: Honoraria|Vault Health: Grant/Research Support **Jane A. O'Halloran, MD PhD**, Janssen Scientific Affairs, LLC: Grant/Research Support **Michael A. Maldonado, MD**, Bristol Myers Squibb: Employee|Bristol Myers Squibb: Stocks/Bonds **William G. Powderly, MD**, Merck: Advisor/Consultant.

